# Vaccination by microneedle patch with inactivated respiratory syncytial virus and monophosphoryl lipid A enhances the protective efficacy and diminishes inflammatory disease after challenge

**DOI:** 10.1371/journal.pone.0205071

**Published:** 2018-10-26

**Authors:** Soojin Park, Youri Lee, Young-Man Kwon, Young-Tae Lee, Ki-Hye Kim, Eun-Ju Ko, Jae Hwan Jung, Manki Song, Barney Graham, Mark R. Prausnitz, Sang-Moo Kang

**Affiliations:** 1 Center for Inflammation, Immunity & Infection, Institute for Biomedical Sciences, Georgia State University, Atlanta, GA, United States of America; 2 School of Chemical and Biomolecular Engineering, Georgia Institute of Technology, Atlanta, GA, United States of America; 3 International Vaccine Institute, Seoul, Korea; 4 Vaccine Research Center, National Institute of Infectious Diseases, National Institutes of Health, Bethesda, MD, United States of America; University of Georgia, UNITED STATES

## Abstract

Intramuscular (IM) vaccination with formalin-inactivated respiratory syncytial virus (FI-RSV) failed in clinical trials due to vaccine-enhanced respiratory disease. To test the efficacy of skin vaccination against respiratory syncytial virus (RSV), we investigated the immunogenicity, efficacy, and inflammatory disease after microneedle (MN) patch delivery of FI-RSV vaccine (FI-RSV MN) to the mouse skin with or without an adjuvant of monophosphoryl lipid A (MPL). Compared to IM vaccination, MN patch delivery of FI-RSV was more effective in clearing lung viral loads and preventing weight loss, and in diminishing inflammation, infiltrating immune cells, and T helper type 2 (Th2) CD4 T cell responses after RSV challenge. With MPL adjuvant, MN patch delivery of FI-RSV significantly increased the immunogenicity and efficacy as well as preventing RSV disease as evidenced by lung viral clearance and avoiding pulmonary histopathology. Improved efficacy and prevention of disease by FI-RSV MN with MPL were correlated with no sign of airway resistance, lower levels of Th2 cytokines and infiltrating innate inflammatory cells, and higher levels of Th1 T cell responses into the lung. This study suggests that MN patch delivery of RSV vaccines to the skin with MPL adjuvant would be a promising vaccination method.

## Introduction

Respiratory syncytial virus (RSV) belongs to the pneumoviridae family [1] and is the leading cause of severe respiratory disease in young children, immunocompromised patients, and the elderly [2, 3]. The hospitalization peaks between 2 and 3 months of age, and severe RSV disease often occurs until 5 years of age [4]. RSV is responsible for recurrent hospitalizations over 3 million admissions and mortality between 66,000 and 190,000 annually and globally in children < 5 years old [5, 6]. Substantial increased mortality happens in older adults with underlying disease following RSV infection at a comparable frequency of influenza [3]. The main target populations for vaccination are young infants and the elderly as well as maternal immunization of pregnant women to prevent severe disease and subsequent complications.

There is no licensed RSV vaccine. Formalin-inactivated whole RSV vaccine (FI-RSV) was tested in clinical trials in children in the 1960s. During the winter season following FI-RSV vaccination, disease was very severe with 80% hospitalization rate and 2 deaths in the vaccinated children less than 2 years of age [7, 8]. FI-RSV vaccine enhanced disease after vaccination and challenge has been extensively reported in different animal models including mice [9], cotton rats [9], cattle [10], and African green monkeys [11]. Inflammatory disease was abrogated in FI-RSV immunized mice that were depleted of CD4 T cells prior to RSV challenge, indicating the critical roles of CD4 T cells in enhancing RSV disease in mice [9]. Toll-like receptor (TLR) agonist adjuvants such as monophosphoryl lipid A (MPL) were previously reported to modulate liposome RSV vaccine immune responses lessening lung inflammation after challenge [12]. RSV vaccine-enhanced disease is a concern for inactivated vaccines administered to infants but was not reported for older adults or older children.

Microneedle (MN) patches contain micron-scale, solid needles that are coated with vaccines in dry formulation, which can be applied to the skin as a patch and administered by minimally trained personnel in a simple and painless manner [13–16]. Previous studies have shown that MN patch vaccination can induce stronger, broader and longer-last immune response than IM vaccination by targeted vaccine delivery to dendritic cells resident in the skin [17–20]. A recent phase 1 clinical trial shown that influenza vaccination by MN patch was safe, immunogenic and well accepted by study participants [21, 22].

RSV vaccination by MN patch has not been studied yet. Delivery of RSV vaccines to the skin via a MN patch would be highly attractive for children who have needle-phobia of intramuscular (IM) needle injection. Also, MN patch vaccination would induce a different profile of immune responses that could be more effective in preventing RSV vaccine-enhanced disease due to targeted skin dendritic cells. FI-RSV would provide a good model antigen to test whether MN delivery of RSV vaccines will diminish RSV vaccine-enhanced disease. In an effort toward administrating RSV vaccines more safely, we hypothesized that MN patch delivery of FI-RSV vaccine to the skin would diminish FI-RSV vaccination-enhanced disease after challenge compared to an IM route in a mouse model. Also, we tested whether FI-RSV MN patch vaccination with MPL adjuvant would increase RSV MN patch vaccine efficacy as well as effectively suppress immune responses prone to causing RSV disease.

## Material and methods

### Mice and virus

Six- to eight-week old BALB/c wild type mice were purchased from Charles River Laboratories International (Wilmington, MA). All animal studies were conducted according to the guidelines of Georgia State University (GSU) Institutional Animal Care and Use Committee (IACUC). RSV A2 strain was originally obtained from Dr. Barney Graham and prepared as described previously [23].

### Cells, antibodies, and reagents

HEp-2 cells were purchased from the American Type Culture Collection (ATCC, Rockville, MD, USA) and maintained in Dulbecco’s modified Eagle’s medium (DMEM; GIBCO-BRL, Grand Island, NY) with 10% fetal bovine serum (FBS, GIBCO-BRL), 2 mM glutamine, penicillin and streptomycin (GIBCO-BRL) at 37°C with 5% CO2. Mouse anti-RSV F monoclonal antibody (131-2A) was purchased from Millipore (Billerica, MA, USA). The horseradish peroxidase (HRP) conjugated goat anti-mouse immunoglobulin G (IgG), IgG1, and IgG2a were from Southern Biotech (Birmingham, AL, USA). Monophosphoryl lipid A (MPL) was purchased from Sigma-Aldrich. RSV F purified protein was prepared in a post-fusion conformation (VRC 3089 pAH RSV A2 F dF) and provided by Vaccine Research Center (VRC), National Institute of Infectious Diseases (NIAID), National Institutes of Health (NIH), Bethesda, MD 20892, USA. RSV A2 G protein fragment (aa131-230) was prepared as previously described [24].

### Preparation of formalin-inactivated RSV (FI-RSV) and vaccine coating on microneedles

FI-RSV was prepared by a slightly modified protocol as described previously [25]. In brief, RSV (A2) infected Hep2 cells were cultured by media for 3 to 5 days at 37°C. The cells and media were collected to cold chilled tubes from the RSV infected Hep2 cell culture flask. The infected cells were sonicated and clarified by centrifugation (2000 x g, 10min, 4°C). Collected supernatants were mixed with filtrated formalin to final concentration (1:4000 vol/vol of 37% formalin) and incubated for 3 days at 37°C with stirring. Inactivated RSV was precipitated from formalin treated supernatants by ultra-centrifugation (80,000–100,000 x g, 1hr, 4°C). The RSV precipitates were suspended with filtered 1xPBS for storage and kept at -80C. Inactivation was confirmed by the absence of plaque forming units (PFU). Each stainless steel MN measured 700 μm in length, 75 μm in thickness and 160 μm in width at the base, which tapered to a sharp tip for vaccine delivery to skin, and were prepared as five-MN linear arrays (Tech Etch, Plymouth, MA). Formalin inactivated (FI)-RSV, 8.7 mg/mL concentration, was mixed with a formulation optimized for MN coating. The optimized formulation consisted of 1% (w/v) carboxymethylcellulose sodium salt (Sigma-Aldrich, St. Louis, MO), 0.5% (w/v), Lutrol F-68NF (BASF, Mt. Olive, NJ), and 15% (w/v) trehalose (Sigma-Aldrich) in phosphate-buffered saline (PBS) [26, 27]. Microneedles (MNs) were coated using an automated dip-coating apparatus [26], and dried in a desiccator at room temperature for 1 day before use. The amount of vaccine coated onto MNs was determined by measuring the protein concentrations in vaccine samples dissolved off MNs using a bicinchoninic acid assay kit (ThermoFisher Scientific, Waltham, MA).

After coating onto MNs, the FI-RSV vaccine retained 85% of its antigenic activity as determined by reactivity of FI-RSV dissolved off MNs in PBS for RSV fusion (F) protein specific monoclonal antibody palivizumab (kindly provided by Dr. Frances Eun-Hyung Lee, Emory University, Atlanta, GA) (S1 Fig). Coated MN arrays each contained 1.1 ± 0.15 μg (proteins) of FI-RSV vaccine.

### MN patch immunization, RSV challenge, and sample collection

Depilatory cream (Nair, Princeton, NJ) was applied on the skin and hair on the dorsal surface of mice was removed for MN patch delivery of vaccines to the clean skin as previously described [17, 28]. Release efficacy of coated vaccines off from MN patches was shown to be over 90% within 2–3 minutes [29]. After 10 minutes in place, residual vaccines in MN patches were below the detection limit [17, 28]. Two patches each containing an array of five MNs coated with FI-RSV was inserted into the skin of each mouse and left in place for 10 min to release the FI-RSV antigens from the coated MNs. There were four experimental vaccine groups: BALB/c mice (n = 5) were immunized one time using (i) FI-RSV vaccine-coated MN patches (2.2 μg) on the skin (FI-RSV MN), (ii) FI-RSV vaccine-coated MNs (2.2 μg) plus MPL (1 μg) adjuvant injected intradermally at the same site (FI-RSV MN+MPL), (iii) intramuscularly (IM) with FI-RSV (2.2 μg) alone dissolved off from MN patches (FI-RSV IM) but was not in alum adjuvant, and (iv) FI-RSV (2.2 μg) plus MPL (1 μg) adjuvant injected intramuscularly (IM+MPL). The phosphate buffered saline (PBS) is a control of unimmunized mice.

Blood samples were collected before and at 2 weeks after single immunization. Unimmunized naive, immunized mice were intranasally challenged with RSV A2 (5×10^5^ PFU in 50 μl per mouse) under isoflurane anesthesia at 3 weeks after single immunization and body weight changes monitored. Individual samples such as lung and bronchoalveolar lavage fluids (BALF) were collected at 5 days post-challenge after sacrifice of mice. Animal experimental procedures were approved and performed by following the guidelines of Georgia State University Institutional Animal Care and Use Committee.

### Measurement of airway hyperresponsiveness with methacholine

Mice were analyzed to assess the airway hyperresponsiveness (AHR) to methacholine (Sigma, St Louis, MO) 4 days after RSV challenge. The mice were placed in a barometric plethysmographic chamber (EMKA Technologies, France) and baseline readings were obtained for 3 min. The enhanced pause (Penh) was calculated according to the manufacturer’s protocol [i.e., (expiratory time/relaxation time-1) (peak expiratory flow/peak inspiratory flow)]. Methacholine was aerosolized into the plethysmographic chamber containing individual mice and maintained for 4 to 7 min until a stable range of values was obtained. The next dose escalation was carried out after 5–8 min. Penh is a dimensionless parameter that represents a function of the proportion of the maximal inspiratory box pressure signals and a function of the timing of expiration. The results are expressed as percentage increases in Penh following the exposure to methacholine (0, 50, and 100mg/ml). The AHR data were presented as the percent increases above the baseline Penh measurements [30, 31].

### ELISA assay for antibody and cytokines responses

Virus-specific antibodies were determined in samples by enzyme-linked immunosorbent assay (ELISA) as previously described [23, 31]. Briefly, FI-RSV (4 μg/ml) was used as a coating antigen. The antibody responses were detected using the secondary antibodies of horse radish peroxidase-conjugated goat anti-mouse IgG, IgG1, and IgG2a (Southern Biotechnology). Antibody concentrations were quantified using the standard curve for each IgG isotype antibodies. The levels of interleukin-4 (IL-4) and IL-5, IL-6, interferon (IFN)-γ and tumor necrosis factor (TNF)-α (eBioscience, SanDiego, CA) in lung extracts and bronchoalveolar lavage fluid (BALF) homogenates were measured using cytokine ELISA kits (eBioscience, SanDiego, CA).

### RSV immuno-plaque assay and RSV neutralizing activity

RSV neutralizing titers and lung viral titers were determined using an immuno-plaque assay (IPA) as described [23]. The serially diluted lung homogenates were added into the monolayer HEp-2 cells and adsorbed for 2 h, then overlaid with growth medium containing 0.8% low temperature gelling agarose prior to incubation for 3 to 4 days. After fixation in formalin, the plaques were detected using the primary anti-RSV F monoclonal (131-2a) antibody, secondary anti-mouse IgG antibody- horse radish peroxidase (HRP) conjugate. As for HRP substrate, 3,3'diaminobenzidine (0.5 mg/ml DAB, 0.01% H_2_O_2_) was used to develop color. The viral titer detection limit is approximately 40 PFU from lung samples in this assay [23].

For the antibody neutralizing assay, immune sera were inactivated at 56°C 30 min and then 2-fold serially diluted in serum-free DMEM. An equal volume of virus (200 PFU in 50 μl) was mixed with serum samples and incubated for 1h. Then the mixture or virus alone (as a positive control) was added to the confluent monolayers of HEp-2 cells and cultured for 3 to 4 days to allow RSV plaque formation. Neutralizing antibody titers were defined as the reverse of serum dilution factors resulting in 50% plaque reduction.

### Flow cytometry

BALF were harvested by infusing 1 ml of PBS into the lungs via the trachea using a 25-gauge catheter to collect cells in the airways at day 5 post-challenge. Lung tissues were homogenized and lung cells were isolated by percoll gradient (44 and 67%) centrifugation. Lung cells were stimulated with G peptide (G_183–195_ CD4 T cell epitope, 4 μg/ml) or F peptide (F_85–93_ CD8 T cell epitope, 4 μg/ml) for 5 h. After stimulation in the presence of peptides, lung cells were stained with intracellular cytokine antibodies and then the cells were fixed and permeabilized according to the manufacturer’s instructions (BD Biosciences). Intracellular cytokines and surface markers for infiltrating cell phenotypes or T cells were stained with antibodies for IFN-γ, IL-4, IL-13, TNF-α (BioLegend), CD45 (clone 30-F11), CD11b (clone M1/70), CD11c (clone N418), F4/80 (clone BM8), Ly6c (clone HK1.4), MHC class II (clone M5/114.15.2), Siglec F (clone E50-2440), B220 (clone RA3-6B2), CD103 (clone 2E7), CD3 (clone 17A2), CD4 (clone GK1.5), and CD8 (clone 53–6.7) as previously described [32]. For analysis, the Becton-Dickinson LSR-II/Fortessa flow cytometer (BD, San Diego, CA) was used to collect cell populations. Acquired flow cytometry data were analyzed by using Flowjo software (Tree Star Inc.).

### Lung histopathology

The lungs collected at 5 day after challenge were fixed with 10% formalin in PBS. After fixation, the tissues were transferred to 70% alcohol and processed using a Shandon Excelsior AS (Thermo Fisher Scientific) and a standard program of dehydration. Finally, the tissue was soaked in paraffin for 2×30 min and then in the second paraffin bath for at least 1 h. Lung tissue sections were stained with hematoxylin and eosin (H&E) and periodic acid-schiff stain (PAS) to assess histopathological changes and mucin expression respectively as described previously [31, 33]. Numerical assessment of histopathology scoring was based on a scale of 0–5 by blinded observers with the severity scoring system similar to the one as previously described [34]. Based on an ordinal scale, a score “<1” is represented within a normal or naive range whereas a maximum score of “5” is represented as extensive or severe histopathologic changes and infiltrates [34] (lymphocytes, polymorphonuclear cells, macrophages, eosinophils) in peribronchiolar, perivascular, interstitial, and alveolar spaces [31, 33]. The mucin expression was detected with PAS-positive area.

### Statistical analysis

Statistical differences were performed using GraphPad software. Data were analyzed for significance using one-way ANOVA with Tukey’s test for multiple comparisons. The difference was considered statistically significant when the P value was less than 0.05.

## Results

### FI-RSV MN patch vaccination with MPL increases RSV specific antibodies

It is important to determine the antigenic stability of MN patch vaccines prior to the assessment of MN patch vaccine efficacy since MN patch coating process might affects the FI-RSV vaccine antigenic stability. FI-RSV vaccine dissolved off from MN patches in PBS was found to maintain antigenic stability at a similar level to that of FI-RSV vaccine stored as a liquid at 4°C as determined by reactivity to palivizumab monoclonal antibody specific for F protein (S1 Fig). Palivizumab is known to recognize the epitope in the site II of RSV F proteins, which is present in both pre-fusion and postfusion conformation of F proteins and FI-RSV. This result is consistent with a recent study demonstrating that motavizumab (recognizing similar epitopes as Palivizumab) strongly binds to FI-RSV, suggesting that FI-RSV well exposes the site II epitope [35].

**Fig 1:**
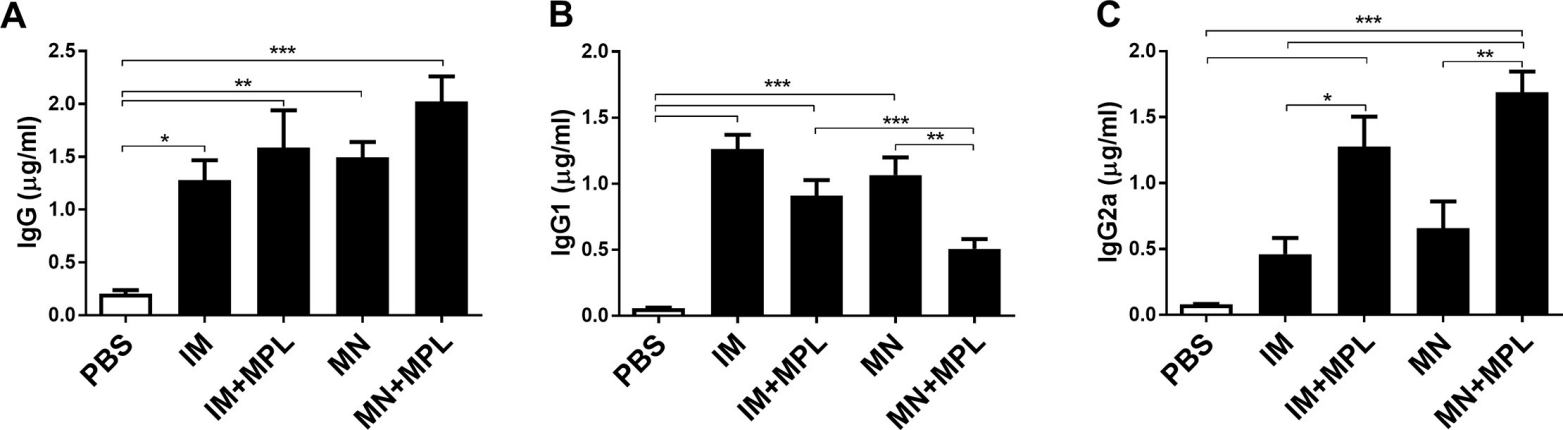
RSV specific IgG and isotype IgG antibody responses after prime immunization with FI-RSV-via IM or MN patch. RSV specific IgG, isotype IgG1 and Ig2a serum antibodies were determined at 2 weeks after prime immunization (n = 5) by ELISA using FI-RSV as a coating antigen. (A) IgG, (B) IgG1 and (C) IgG2a levels. IM: intramuscular (IM) immunization with FI-RSV (2.2 μg), IM+MPL: IM immunization with FI-RSV (2.2 μg) + MPL (1 μg), MN: MN (microneedle) patch immunization in skin with FI-RSV (2.2 μg), MN+MPL: MN patch immunization with FI-RSV (2.2 μg) + MPL (1 μg) injected intradermally. RSV specific IgG concentrations (μg/ml) are presented as mean ± SEM (n = 5). The experiments were performed in duplicates. PBS: Unimmunized (no vaccine in PBS) control. Statistical significances were performed by one-way ANOVA and Tukey’s multiple-comparison tests in GraphPad Prism; *** p<0.001, **p<0.01, *p<0.05.

To determine the immunogenicity and efficacy of MN patch FI-RSV vaccines, groups of mice (n = 5) were immunized with MN patch FI-RSV (2.2 μg) vaccine on the skin with (MN+MPL, Fig 1) or without MPL adjuvant injected intradermally or were intramuscularly (IM) immunized with FI-RSV (2.2 μg) as a single dose vaccination strategy (Fig 1). RSV specific IgG antibodies (Fig 1A) were observed at significantly higher levels in the vaccinated groups with the MN+MPL group being the highest, compared to the unvaccinated control (PBS). A pattern of IgG isotypes indicates the T helper types 1 (IgG2a) and 2 (IgG1) of T cell responses [36, 37]. The IM+MPL and MN+MPL groups induced higher levels of IgG2a and lower levels of IgG1 antibodies specific for RSV compared to the IM and MN groups, which are statistically significant (Fig 1B and 1C).

**Fig 2:**
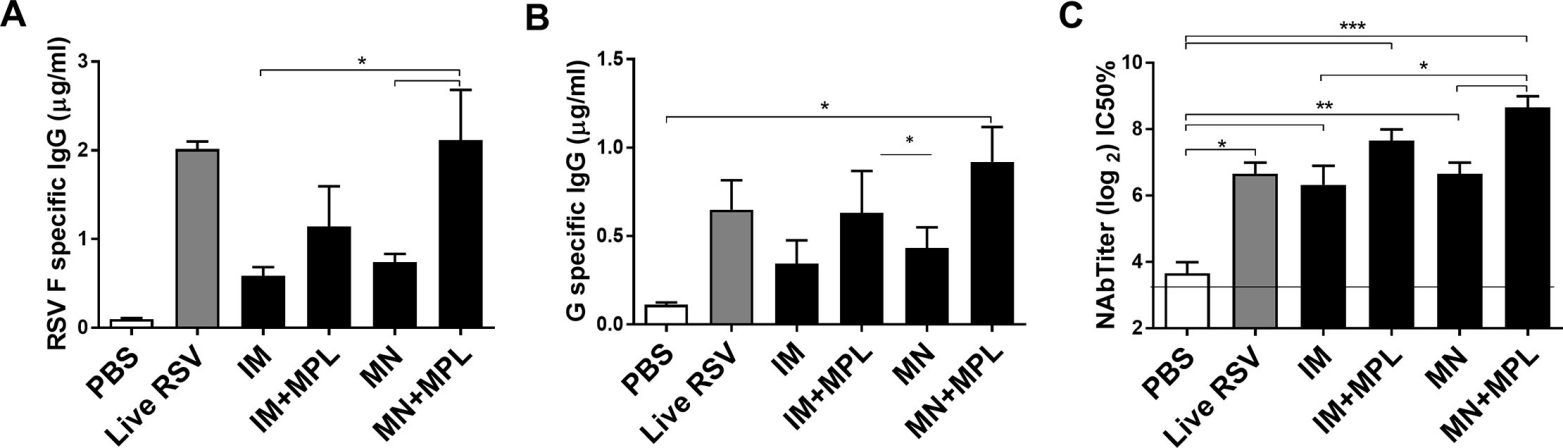
IgG antibody responses specific for F and G protein antigens, and RSV neutralizing titers in prime immune sera. RSV F and G protein specific IgG antibodies were determined by ELISA in sera collected at 3 weeks after prime immunization of mice (n = 5). (A) F protein specific IgG antibodies (μg/ml). (B) IgG antibodies (μg/ml) specific for G_130-230_ fragment. (C) RSV neutralizing antibody titers. RSV neutralizing antibody titers were determined by 50% plaque forming reduction (IC50%) assay. The linear line represents a lower limit of detection in RSV neutralizing titers. Results are presented as mean ± SEM (n = 5) and representative of duplicate experiments. Live RSV: the group of mice that were intranasally inoculated with RSV A2 (1x10^4^ PFU/50 μl) as a live RSV control. Other groups are the same as described in the legend Fig 1. Statistical significances were performed by one-way ANOVA and Tukey’s multiple-comparison tests in GraphPad Prism or student T-test; ***p<0.0001, **p<0.001, *p<0.05.

When IgG antibody levels were determined by using purified F proteins and G protein fragment, the MN+MPL group showed highest levels of IgG antibodies specific for RSV F and G proteins, which are comparable to those in live RSV sera and significantly higher than those in the MN group (Fig 2A and 2B). We also determined the titers of RSV neutralizing antibodies that are important for protection against RSV. Highest titers of RSV neutralizing antibodies were detected up to 8.5 of Log2 in sera from the FI-RSV MN+MPL group, which are significantly higher than those in the MN patch and IM groups (Fig 2C). The FI-RSV MN patch group showed moderately higher titers of RSV neutralizing activity than the live RSV group although the difference was not significant (Fig 2C).

### A single dose of FI-RSV MN patch vaccination confers effective protection against RSV

**Fig 3:**
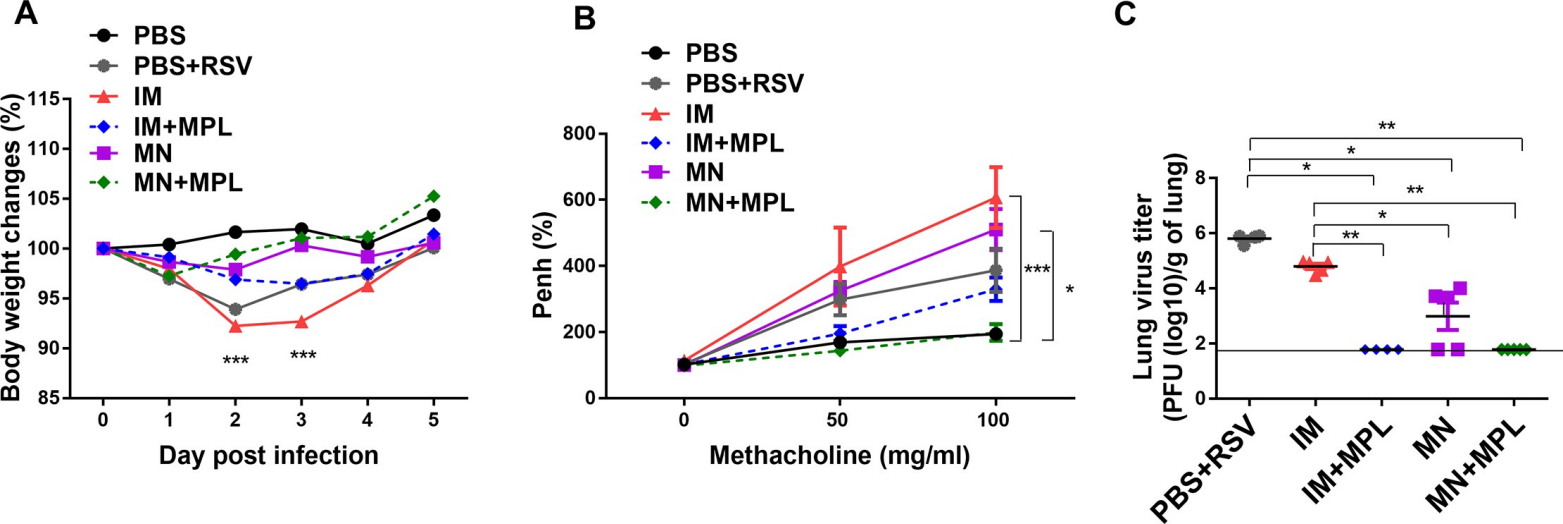
A single dose of FI-RSV MN patch delivery to the skin confers effective protection against RSV without disease. Vaccinated and naïve mice (n = 5) were intranasally challenged with RSV A2 (5×10^5^ PFU) at 3 weeks after a single dose. (A) Body weight changes after RSV infection. (B) Airway hyper-responsiveness to methacholine. PenH (enhanced pause) was measured by a whole body plethysmograph in live mice at day 4 post RSV challenge, with increasing concentrations of inhaled methacholine (0–100 mg/ml). (C) Lung RSV titers day 5 post challenge. The viral titers were confirmed in duplicate experiments and the dotted line represents the detection limit. PBS: unimmunized mice. PBS+RSV: unimmunized mice with RSV infection. The groups of IM, IM+MPL, MN, and MN+MPL are the same as described in Fig 1. Results are presented as mean ± SEM. Statistical significances were performed by one-way ANOVA and Tukey’s multiple-comparison tests in GraphPad Prism; *** p<0.001, **p<0.01, *p<0.05.

To determine the efficacy of protection against RSV, immunized mice were challenged with RSV at 3 weeks after a single dose (Fig 3). The FI-RSV IM group showed significant weight loss (~8.5%) followed by the naïve infection group with 6.5% average weight loss (Fig 3A). Notably, FI-RSV MN skin vaccination resulted in preventing weight loss within 2–3% changes similar to the FI-RSV MN+ MPL skin vaccinated mice with a better recovery. As a measure of breathing difficulty in the lung function, airway resistance enhanced pause values (PenH) were determined by plethysmography and presented in percentages compared to naïve mice (Fig 3B). The FI-RSV MN+ MPL group displayed the lowest PenH similar to the uninfected PBS control group whereas the FI-RSV IM group showed about a 3–4 fold higher PenH values (Fig 3B). FI-RSV IM+MPL immunized mice showed an increase in Penh values by 2 fold compared to FI-RSV MN+ MPL skin vaccinated mice. Naïve mice with infection and FI-RSV MN also exhibited increases in PenH by 3-fold compared to MN+MPL immune mice.

Lung viral titers were determined to assess the protective efficacy day 5 post challenge (Fig 3C). Highest lung viral titers (10^6^ PFU / g lung) were detected in the naïve mice with infection. In contrast, FI-RSV MN+ MPL skin vaccination was found to be most effective in clearing lung viral loads below the detection limit. Also, FI-RSV IM+MPL vaccinated mice showed significantly lower levels of RSV lung viral loads than FI-RSV IM mice (Fig 3C). FI-RSV MN skin delivery was more effective in lowering lung viral titers than conventional IM vaccination (Fig 3C).

**Fig 4:**
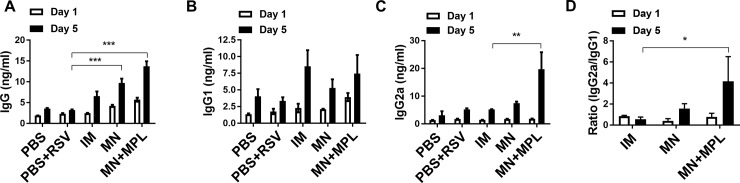
*In vitro* IgG antibody secreting cell responses in splenocytes day 5 post challenge. The cells from the spleens were cultured for 1 day or 5 days and RSV specific IgG antibodies secreted into culture supernatants determined by ELISA. (A) IgG, (B) IgG1, (C) IgG2a and (D) Ratios of IgG2a/IgG1 isotypes levels were analyzed by ELISA using FI-RSV as a coating antigen. Mouse groups are the same as described in Figs 1 and 3. Results are presented as mean (n = 5) ± SEM and were obtained from duplicate experiments. Statistical significances were performed by one-way ANOVA and Tukey’s multiple-comparison tests in GraphPad Prism; *** p<0.001, *p<0.05.

Spleen cells collected day 5 post challenge were *in vitro* cultured to determine IgG antibody secreting cell responses as a measure of B cells rapidly differentiating into plasma cells (Fig 4). The FI-RSV MN+MPL group showed significantly higher levels of RSV specific IgG and IgG2a antibodies secreted into the splenocyte culture supernatants compared to the IM group (Fig 4A and 4C), resulting in higher ratios of IgG2a/IgG1 antibodies (Fig 4D). These results suggest that FI- RSV MN skin immunization enhances IgG2a isotype antibody secreting cell responses.

### FI-RSV MN patch delivery with MPL attenuates histopathology against RSV

**Fig 5:**
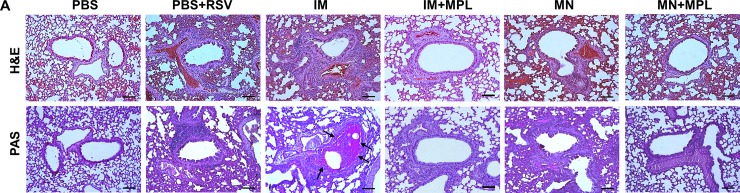
Histopathological changes in lungs from mice after RSV challenge. Lung tissues (n = 5 per group) were collected from individual mice and tissue section were stained with **(A)** hematoxylin and eosin (H&E) and **(B)** periodic acid-Schiff (PAS) to assess pulmonary histopathologic changes day 5 post challenge. Scale bars represent 100 μm (×100 magnification). Mouse groups are the same as described in Fig 3.

**Fig 6:**
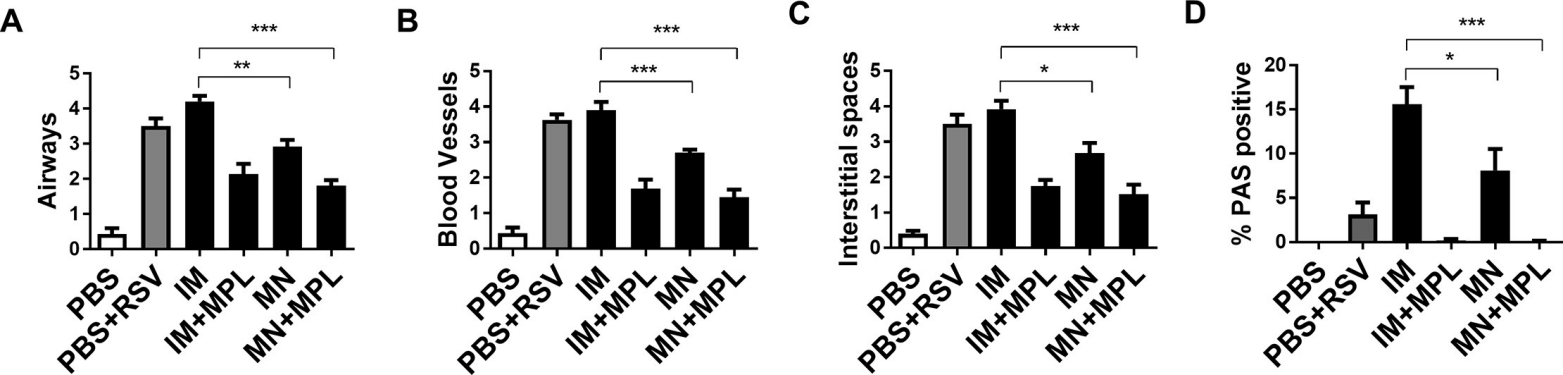
Inflammation scores of lung tissue histopathology after RSV challenge. Inflammation scores were blindly determined by examining the histology slides under the microscope as shown in the Fig 5. FI-RSV MN patch delivery with MPL attenuates histopathology after RSV challenge. (A) Inflammation scores around the airways. (B) Inflammation scores around blood vessels. (C) Inflammation scores around interstitial spaces. (D) Stained lung sections were scored for bronchiolar mucus production as the percentages of PAS positives. Results (n = 5) are presented as mean ± SEM. Statistical significances were performed by one-way ANOVA and Tukey’s multiple-comparison tests in GraphPad Prism; *** p<0.001, **p<0.01, *p<0.05.

To determine the impact of FI-RSV MN vaccination on pulmonary histopathology of mice after challenge, tissue sections were examined after staining with H&E and PAS (Fig 5A and 5B). FI-RSV immunized via an IM route resulted in the highest degree of inflammation scores around the airways, blood vessels, and interstitial spaces as well as PAS positive mucus production (Fig 6A–6D). Naïve animals in general exhibit mild pathology after RSV infection. In this study, even the naïve mice after RSV infection displayed moderate to substantial levels of lung histopathology (Figs 5 and 6). The FI-RSV MN skin immunization showed less pulmonary histopathology compared to FI-RSV IM vaccination. FI-RSV IM with MPL adjuvanted vaccination of mice also did not display lung histopathology after RSV challenge (Figs 5 and 6). FI-RSV MN + MPL skin vaccination resulted in the lowest degree of pulmonary histopathology and no PAS positive mucus secretion, less than naïve mice after RSV challenge (PBS+RSV). Taken together, these results provide evidence that MN patch delivery of FI-RSV vaccine reduced lung inflammation compared to IM route and effectively prevented FI-RSV vaccine-enhanced histopathology particularly in the presence of MPL adjuvant.

### FI-RSV MN patch delivery with MPL does not enhance Th2 cytokines in lungs

**Fig 7:**
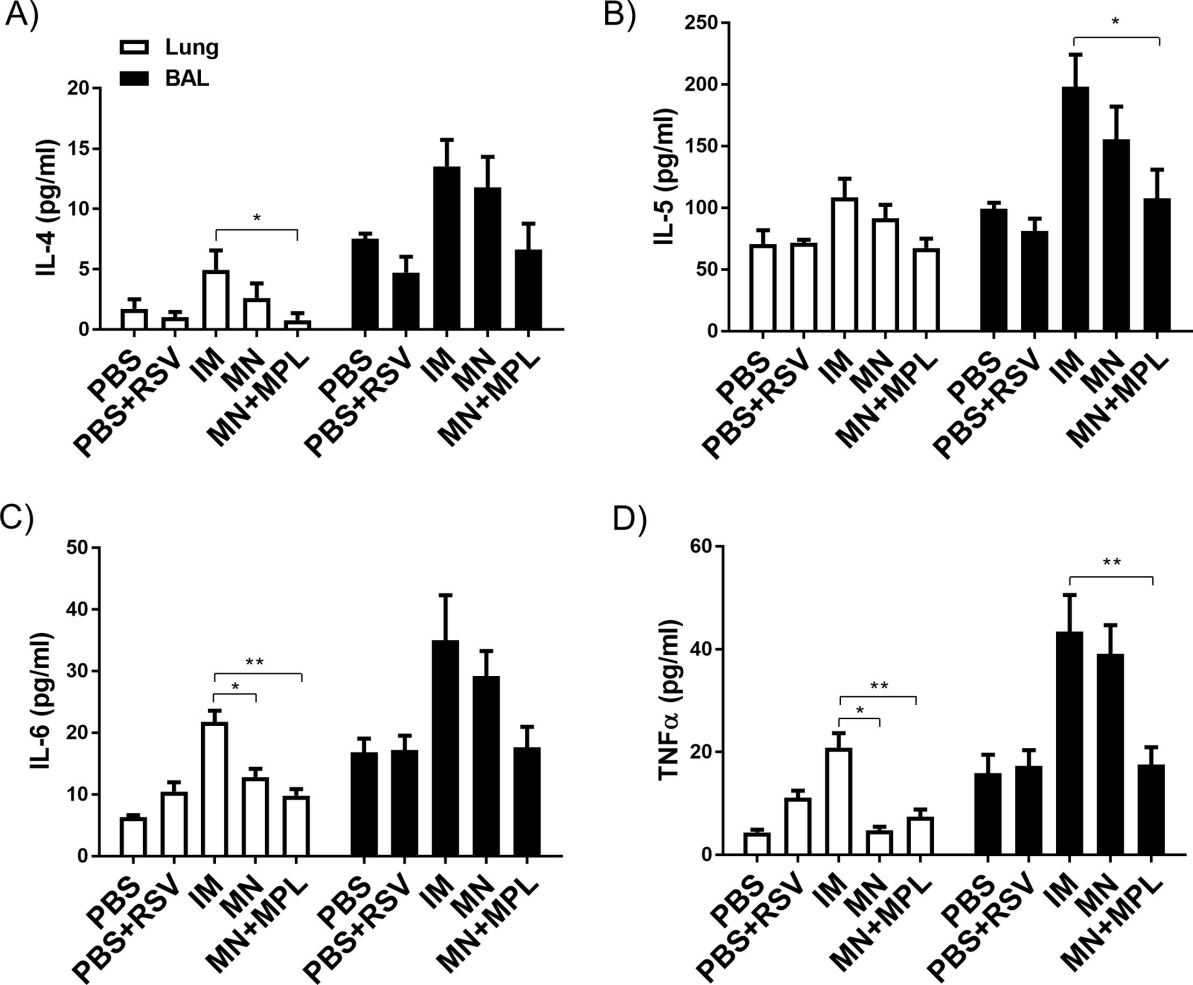
Cytokine levels in lung samples after RSV challenge. The levels of (A) IL-4, (B) IL-5, (C) IL-6 and (D) TNF-α were determined in the lung homogenates day 5 post RSV challenge by a cytokine ELISA. Results (n = 5) are presented as mean ± SEM and representative of duplicate experiments. Statistical significances were performed by one-way ANOVA and Tukey’s multiple-comparison tests or t-tests and nonparametric Mann–Whitney U-test in GraphPad Prism;, **p<0.01, *p<0.05.

Inflammatory cytokines are often associated with RSV vaccination-enhanced disease after RSV challenge. As shown in the cytokine ELISA of lung homogenates, highest levels of Th2 (IL-4, IL-5, IL-6) and TNF-α cytokines, in mice with FI-RSV IM immunization after RSV challenge (Fig 7A–7D). The FI-RSV MN group showed lower levels of IL-6 and TNF-α cytokines in lung samples than the FI-RSV IM group (Fig 7C and 7D). FI-RSV MN + MPL immunization did not increase the levels of inflammatory cytokines, which is close to the levels in naïve mice with or without infection (Fig 7).

### FI-RSV MN delivery with MPL enhances Th1 but reduce Th2 T cell responses in lungs after challenge

**Fig 8:**
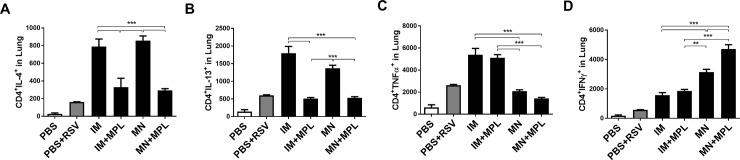
CD4 T cells secreting Th1 or Th2 cytokines in lungs as determined by intracellular cytokine staining. Intracellular cytokine staining of lung cells was carried out by Flow cytometry after *in vitro* stimulation with G_183–195_ peptides, a known CD4 T cell epitope, and followed by staining with CD45 and CD4 surface marker antibodies and intracellularly with cytokine (A) IL-4, (B) IL-13, (C) TNFα, (D) IFN-γ in lung cells. Results (n = 5) are presented as mean ± SEM and representative of duplicate experiments. The Y axis indicate average CD4^+^cytokine^+^ cell numbers per lung per mouse in the groups. Mouse groups are the same as described in Fig 3. Statistical significances were performed by one-way ANOVA and Tukey’s multiple-comparison tests in GraphPad Prism; *** p<0.001, **p<0.01.

Next, we determined CD4 T cells secreting cytokines in the lung samples collected day 5 post challenge using an assay of intracellular cytokine staining after stimulation with a CD4 T cell epitope, G_183–195_ peptides. The FI-RSV IM group showed the highest levels of CD4 T cells producing IL-4, IL-13, and TNFα in lungs (Fig 8). The FI-RSV MN group displayed high levels of IL-4 and IL-13 secreting CD4 T cells but not TNFα+ CD4 T cells (Fig 8A, 8B and 8C). In contrast, the FI-RSV IM+MPL group exhibited low levels of IL-4, IL-13, INF-γ but a high level of TNFα CD4 T cells (Fig 8). The groups of FI-RSV MN + MPL showed lower levels of CD4 T cells secreting Th2 (IL-4, IL-13) and TNFα cytokines but the highest level of Th1 INF-γ CD4 T cells (Fig 8).

**Fig 9:**
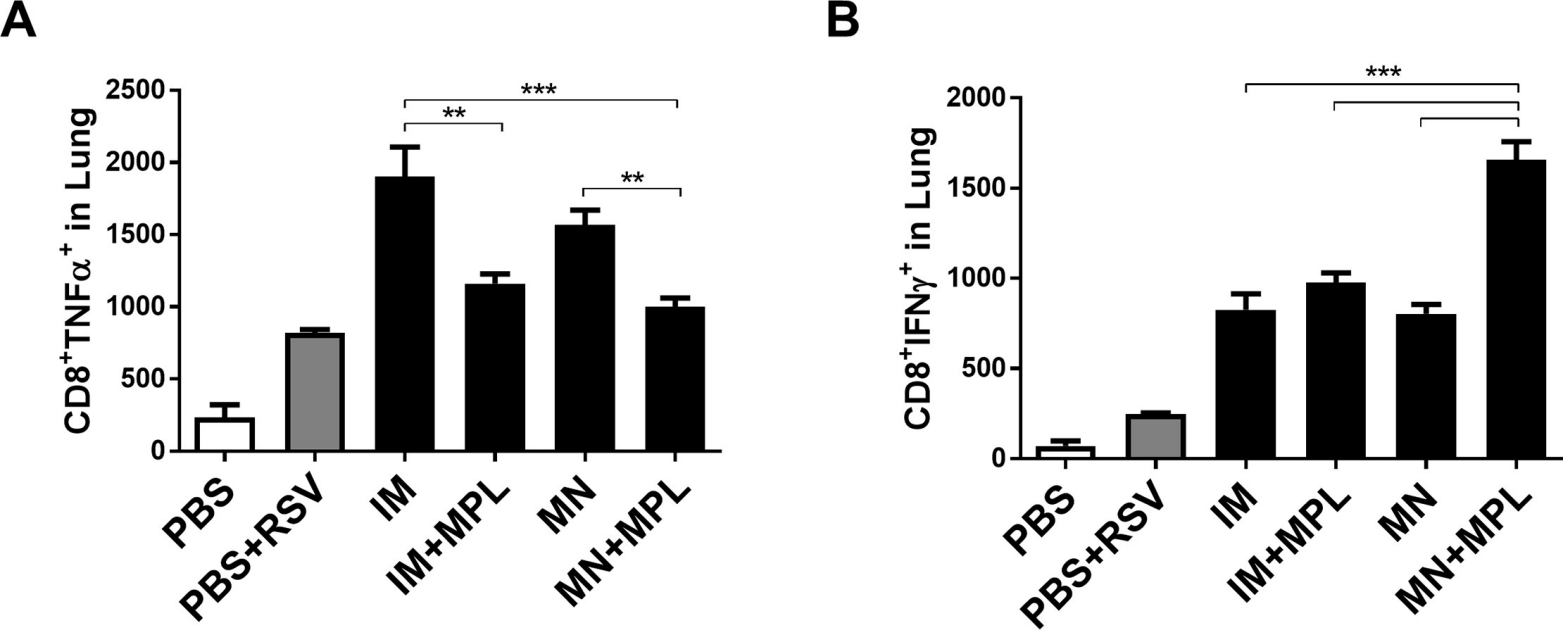
Lung CD8 T cells secreting TNF-α or IFN-γ cytokines as determined by intracellular cytokine staining. Intracellular cytokine staining of lung cells was carried out by Flow cytometry after *in vitro* stimulation with F_85–93_ peptides, a known CD8 T cell epitope, and followed by staining with CD45 and CD8 surface marker antibodies and intracellularly with cytokine antibodies (A) TNF-α. (B) IFN-γ. The Y axis indicate CD8^+^cytokine^+^ average cell numbers per lung per mouse in the groups. Mouse groups are the same as described in Fig 3. The data are representative of duplicate experiments. Results are presented as mean ± SEM. Statistical significances were performed by one-way ANOVA and Tukey’s multiple-comparison tests in GraphPad Prism; *** p<0.001, **p<0.01.

We also analyzed CD8 T cells in the lung samples collected day 5 post challenge, after *in vitro* stimulation with F_85–93_ peptides, a known CD8 T cell epitope, using an intracellular cytokine staining assay (Fig 9). TNFα secreting lung CD8 T cells were detected at higher levels in the FI-RSV IM and MN groups compared to those in the IM+MPL and MN+MPL groups respectively (Fig 9A). In contrast, the MN+MPL group displayed higher level of INF-γ secreting lung CD8 T cells than other vaccine (IM, IM+MPL, MN) groups after challenge (Fig 9B). Overall, these results suggest that MN skin delivery of FI-RSV in the presence of MPL adjuvant promotes the induction of Th1 IFN- γ cytokine secreting CD4 and CD8 T cells as well as suppresses Th2 type (IL-4, IL-13) and pro-inflammatory TNF-α cytokine producing T cell responses.

### FI- RSV MN patch delivery avoids inflammatory cellular infiltrates upon RSV challenge

**Fig 10:**
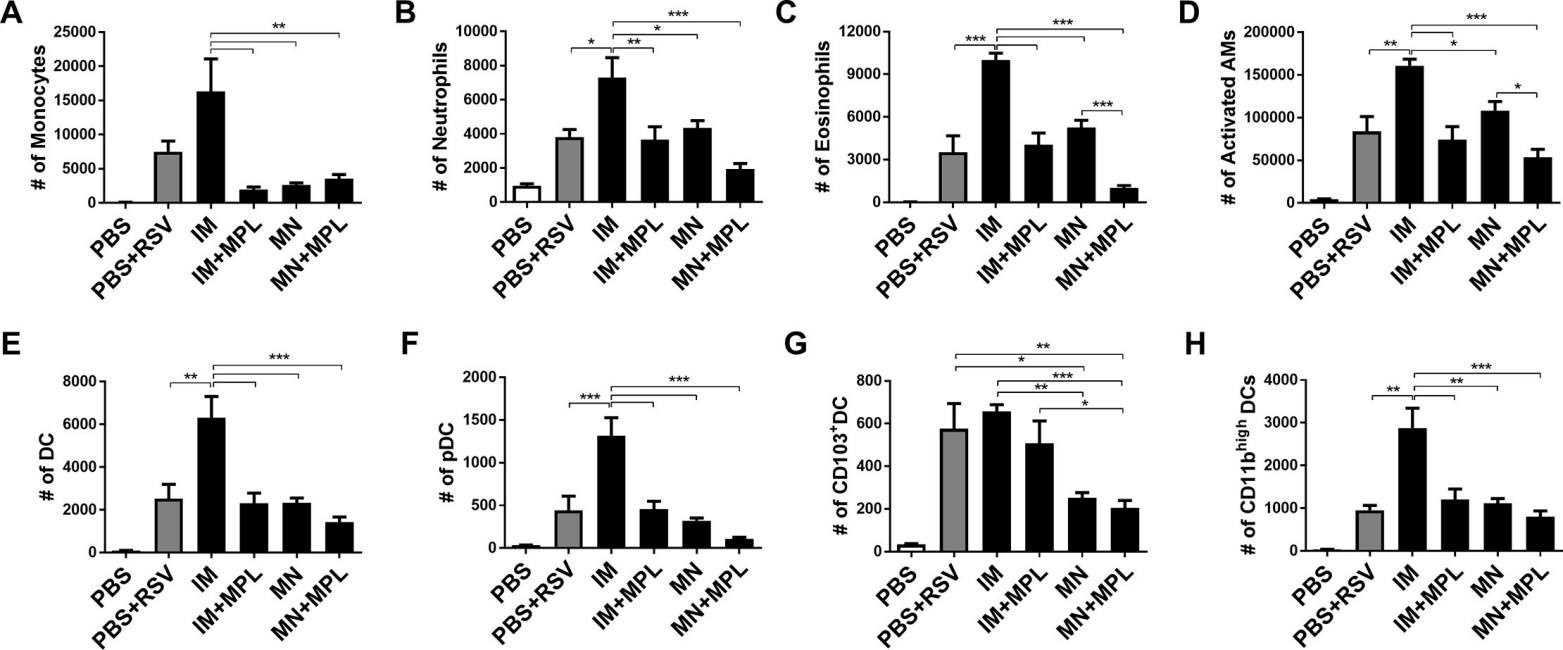
FI- RSV MN patch delivery prevents inflammatory cellular infiltrates into the airway BALF upon RSV challenge. Cells from BALF collected at 5 days after challenge were stained with cell type-specific marker antibodies and analyzed by flow cytometry. Results (n = 5) are presented as mean ± SEM and representative of duplicate experiments. The Y axis indicate average infiltrating each designated phenotypic cell numbers per BALF per mouse in the groups. Mouse groups are the same as described in Fig 3. Statistical significances were performed by one-way ANOVA and Tukey’s multiple-comparison tests in GraphPad Prism; *** p<0.001, **p<0.01, *p<0.05.

**Fig 11:**
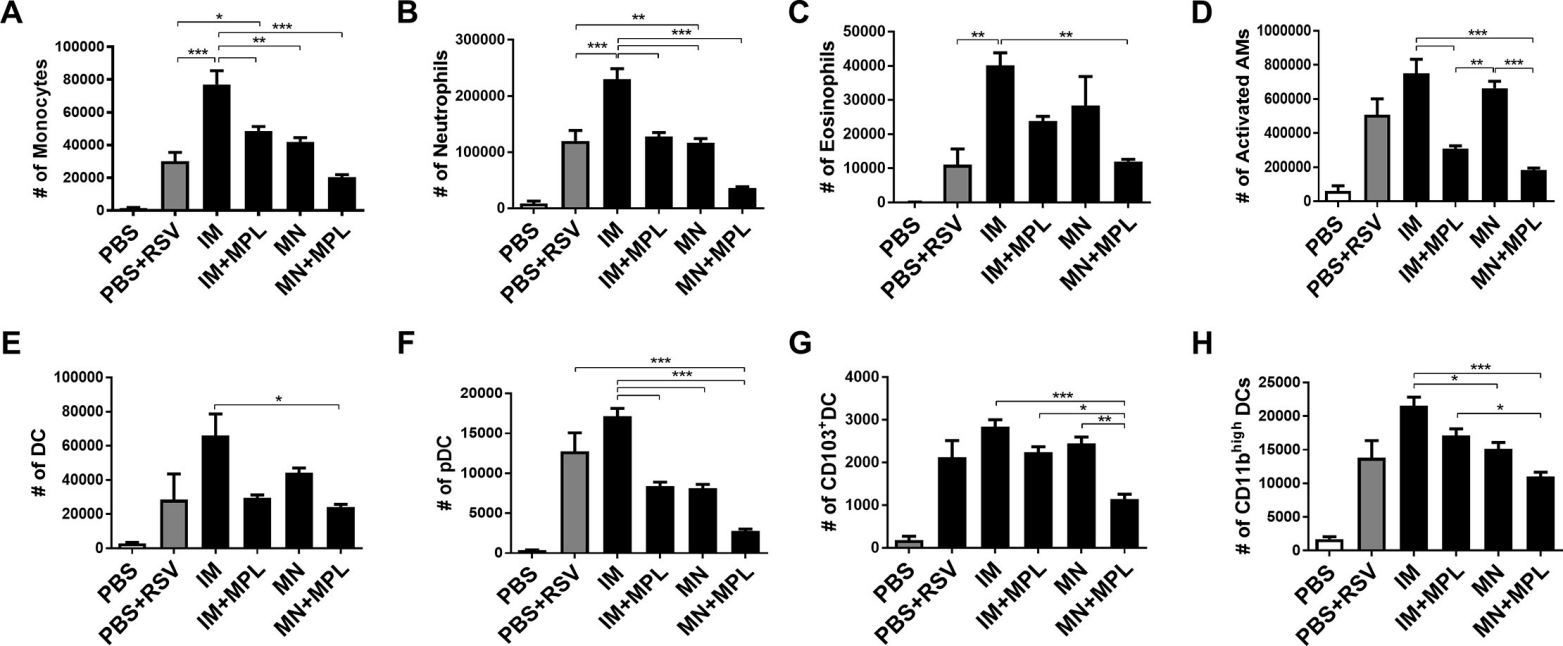
Cellular infiltrates into the lungs are low or prevented by MN patch delivery of FI-RSV after RSV challenge. Cells from lungs collected at 5 days after challenge were stained with cell type-specific marker antibodies and analyzed by flow cytometry. Results (n = 5) are presented as mean ± SEM and representative of duplicate experiments. The Y axis indicate average infiltrating each designated phenotypic cell numbers in the lung tissue per mouse in the groups. Mouse groups are the same as described in Fig 3. Statistical significances; *** p<0.001, **p<0.01, *p<0.05.

To better understand the effects of different routes of FI-RSV vaccine delivery on lung inflammation, we determined the cellular phenotypes. Cells from BAL (Fig 10) and lungs (Fig 11) collected at 5 days after challenge were stained with cell type-specific marker antibodies and analyzed by flow cytometry. The PBS+RSV infection and FI-RSV IM groups showed the highest levels of cellular infiltrates in BAL, which include monocytes (CD11b^+^F4/80^+^Ly6C^high^), neutrophils (CD11b^+^F4/80^-^Ly6C^+^), activated alveolar macrophages (F4/80^+^CD11c^+^MHCII^high^), and DCs (CD45^+^F4/80^-^CD11c^+^MHCII^+^), plasmacytoid DCs (pDC, B220^+^CD11c^+^F4/80-), CD103^+^ DCs (CD11c^+^CD103^+^F4/80^-^), CD11b^+^ DCs (CD11c^+^CD11b^+^F4/80^-^) as shown in Fig 10. Also, the FI-RSV IM group displayed highest levels of infiltrating cells in the lung (monocytes, neutrophils, different subsets of DCs) (Fig 11). The IM+MPL and MN groups showed similarly moderate to lower cellular infiltrates (monocytes, neutrophils, AMs, conventional DCs, pDCs) in BALF and lungs compared to the IM group (Figs 10 and 11). Significantly lowest levels of innate immune cells, particularly monocytes, neutrophils, and activated macrophages were detected in both lung and BALF samples from the MN patch delivery group with MPL (MN+MPL). These data on the cellular phenotypes of infiltrates provide evidence that MN patch delivery of RSV vaccine with MPL adjuvant avoids infiltrates of inflammatory innate immune cells.

## Discussion

The primary target population for RSV vaccination includes young children, maternal immunization of pregnant women to protect the infant, and the elderly. A patch-based skin delivery vaccine would be potentially self-applicable, avoid needle-phobia, and could be safer and more effective. Therefore, patch-based vaccination would have a better acceptability for vaccinating the target population. Clinical trials of injectable FI-RSV IM vaccines failed due to the induction of atypical vaccine-enhanced respiratory disease in vaccinated children after exposure to natural RSV infection [7, 8]. Since FI-RSV vaccination of mice is well known to cause vaccine-enhanced lung inflammation after RSV challenge, we proposed that FI-RSV would serve as a good model antigen to test whether RSV vaccine delivery to the skin in combination with MPL adjuvant would diminish RSV vaccine-associated disease. The immune profiles, efficacy, and histopathology were examined in mice after MN patch vaccination or conventional intramuscular (IM) immunization with FI-RSV in the presence or absence of MPL. The results in this study suggest that the immunogenicity and protective efficacy of RSV vaccines would be improved by FI-RSV MN patch vaccination and that RSV vaccine-associated inflammatory disease could be diminished or preventable by delivering vaccines via MN + MPL adjuvant to the skin or IM + MPL compared to those by convention IM injection.

Retention of vaccine antigenic stability after MN coating of influenza vaccines was shown to be critical for inducing protective immunity after vaccination [26, 38, 39]. The antigenic property of binding to palivizumab was retained in FI-RSV after dissolving off of MN FI-RSV vaccine dry formulation in PBS solution, suggesting the antigenic integrity of FI-RSV MN. FI-RSV was shown to be highly reactive to the site II epitope neutralizing antibody motavizumab (recognizing similar epitopes as Palivizumab) [35]. Lung viral clearance was improved in FI-RSV MN primed mice than that in mice with FI-RSV IM. Compared to FI-RSV IM immunization, delivery of FI-RSV via MN patch in the skin was found to significantly reduce pulmonary inflammatory disease as evidenced by less weight loss and low histopathology, and lower levels of lung IL-6 and TNF-α cytokines. Depletion of CD4 T cells was shown to significantly reduce inflammatory disease after FI-RSV vaccination of mice prior to challenge [9], supporting that Th2 CD4 T cells play a critical role in RSV vaccination-enhanced respiratory disease. Lung and BAL CD4 T cells producing Th2 (IL-4, IL-13) and TNF-α cytokines were at higher levels in FI-RSV MN or IM immune mice than those in IM+MPL and MN+MPL groups, similar to the unimmunized control group with RSV infection. G_183–195_ epitope responsive CD4 T cells were not detected at sufficient levels after an acute RSV infection of naïve mice [40]. Whereas the immunized mice with RSV G expressing vaccinia virus expanded G_183–195_ epitope responsive CD4 T cells primarily Th1 cells and Th2 cells up to 40% of the CD4 T cells in the lungs within 7 days after challenge [40–42]. The study from Johnson et al showed that CD4 T cells from FI-RSV immunized mice responded to a diverse array of peptides after challenge with RSV [43]. FI-RSV immunized mice were shown to induce higher levels of G_183–195_ epitope responsive CD4 T cells secreting IL-4 but not IFN-γ at 5 days after challenge [43]. Our results on IL-4^+^ CD4 T cell responses to G_183–195_ epitope are consistent with that in the Johnson et al study [43]. We observed higher levels of IFN- γ^+^ CD4 T cells responsive to G_183–195_ epitope and IFN- γ^+^ CD8 T cells responsive to F_85–93_ epitope in the MN+MPL group but not in the IM and MN groups. Consistent with less pulmonary inflammation, lung infiltrating immune cells (monocytes, neutrophils, different subsets of DCs) were low in FI-RSV MN mice and these infiltrating cells were at lowest levels in the MN+MPL group compared to FI-RSV IM. Therefore, MN patch RSV vaccination in combination with MPL might provide a promising route for an RSV vaccine candidate.

In the Delgado manuscript [44], a very low dose of FI-RSV (1 x 10^5 plaque forming unit (PFU) to have the same PFU dose of live RSV control) was used to immunize mice, resulting in immune responses of low RSV neutralizing antibodies. The dose of FI-RSV used in the Delgado manuscript is very low dose (estimated to be approximately 0.15 μg FI-RSV proteins) [44]. Nonetheless FI-RSV (2.2 μg) alone IM was not effective in clearing lung viral titers. MPL adjuvant is licensed to be included in human vaccines [45]. MPL adjuvant formulated FI-RSV IM vaccination of cotton rats was previously shown to reduce the induction of cytokines and histopathology but did not improve the clearance of lung viral titers [46]. Soluble RSV F protein vaccines with MPL (15 or 50 μg) were used to immunize cotton rats via heterologous routes of intranasal prime and intradermal boost and found to enhance protection against RSV and to reduce both cytokine levels and lung histopathology, compared to intranasal prime boost [47]. In this study, a single dose of FI-RSV (2.2 μg) MN + MPL (1 μg) significantly enhanced the immunogenicity (IgG, RSV neutralizing titers) and the efficacy of lung viral clearance but showed no evidence of RSV disease (weight loss, PenH, histopathology). Compared to FI-RSV IM, the FI-RSV MN + MPL adjuvant group did not induce CD4 T cells producing Th2 cytokines, infiltrating innate immune cells locally in the lungs and BALF after RSV challenge. A previous study reported that incorporation of MPL adjuvant into RSV virosome vaccines resulted in enhancing immunogenicity and Th1 immune responses, and reducing lung pathology, compared to non-adjuvanted virosome vaccines in mice [12]. MPL adjuvant effects on enhancing the efficacy of FI-RSV IM and reducing histopathology were similarly observed as those presented in the FI-RSV MN + MPL group. In this study focusing on MN delivery, FI-RSV and MPL were delivered separately (i.e., by MN patch and intradermal injection, respectively); future studies should address incorporation of MPL (or other adjuvants) into MN patch formulations for co-delivery of vaccine and adjuvant.

In summary, this study provides evidence that different routes of RSV vaccine delivery may impact the efficacy and vaccine safety. FI-RSV would not be used in naïve infants. Once an RSV vaccine candidate is developed, MN patch vaccination would be a promising route of RSV vaccination potentially improving the efficacy and safety. Intradermal application of MPL adjuvant in the FI-RSV MN vaccination could significantly enhance the immunogenicity and efficacy of FI-RSV MN. Use of subunit RSV vaccines still represents a concern of vaccine-associated disease in naïve young infants. It is expected that the concept and method of FI-RSV MN + MPL vaccination might be applicable for improving subunit vaccine efficacy in children and the elderly populations who might have prior RSV infection.

## Supporting information

S1 Checklist NC3Rs ARRIVE guidelines checklist 2014 (PLOS)(9 14 181). (PDF)

S1 Fig Antigenic stability of FI-RSV after coating onto solid MN. The antigenic stability of FI-RSV coated onto solid stainless MN was determined by reactivity of FI-RSV de-coated and dissolved off from MN in PBS for RSV fusion (F) protein specific monoclonal antibody palivizumab. The data are representative out of 3 multiple tests and we observed reproducibility in coating FI-RSV vaccines onto MN patches and antigenic reactivity of FI-RSV vaccines.
(PDF)
