# Supplementary material for: Vaccination by microneedle patch with inactivated respiratory syncytial virus and monophosphoryl lipid A enhances the protective efficacy and diminishes inflammatory disease after challenge
Source: PLoS One. 2018 Oct 26;13(10):e0205071. doi: 10.1371/journal.pone.0205071 (PMC6203256; doi:10.1371/journal.pone.0205071)
Supplement: S1 Fig — The antigenic stability of FI-RSV coated onto solid stainless MN was determined by reactivity of FI-RSV de-coated and dissolved off from MN in PBS for RSV fusion (F) protein specific monoclonal antibody palivizumab. The data are representative out of 3 multiple tests and we observed reproducibility in coating FI-RSV vaccines onto MN patches and antigenic reactivity of FI-RSV vaccines. (PDF) [file pone.0205071.s002.pdf]

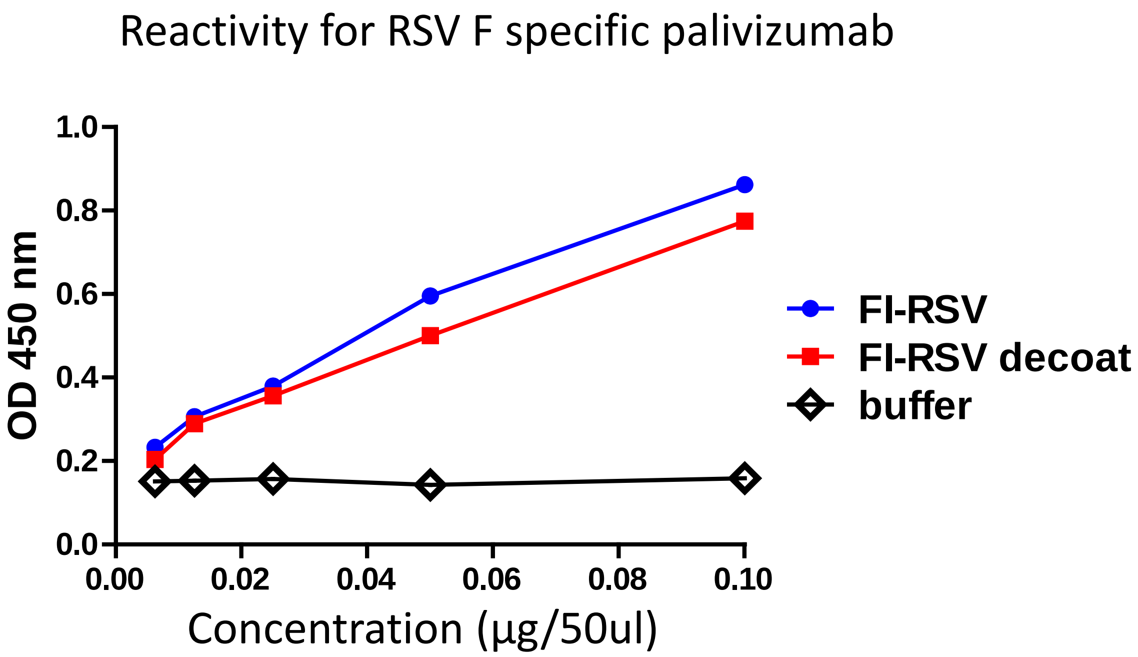

Supplementary Figure S1. Antigenic stability of FI-RSV after coating onto solid MN. The antigenic stability of FI-RSV coated onto solid stainless MN was determined by reactivity of FI-RSV de-coated and dissolved off from MN in PBS for RSV fusion (F) protein specific monoclonal antibody palivizumab. The data are representative out of 3 multiple tests and we observed reproducibility in coating FI-RSV vaccines onto MN patches and antigenic reactivity of FI-RSV vaccines
